# A Coumarin-Based Probe for Sequential ON–OFF–ON Detection of Cu^2+^ and Biothiols: Naked-Eye Detection, Smartphone RGB Readout and In Vivo Imaging

**DOI:** 10.3390/bios16060351

**Published:** 2026-06-22

**Authors:** Mingjie Wei, Linxin Zheng, Weilong Tian, Xingfeng Wang, Rong Liu, Lijuan Chen, Li Niu

**Affiliations:** 1 School of Economics and Statistics, Guangzhou University, Guangzhou 510006, China; 2 School of Chemistry and Chemical Engineering, Guangzhou University, Guangzhou 510006, China; 3 School of Pharmacy, Xianning Medical College, Hubei University of Science and Technology, Xianning 437100, China; 15755904672@163.com (L.Z.); 15549290473@163.com (W.T.); 18754983951@163.com (X.W.); liur97@126.com (R.L.); 4 School of Chemical Engineering and Technology, Zhuhai Campus, Sun Yat-sen University, Zhuhai 519082, China

**Keywords:** sequential detection, detection of Cu^2+^ and biothiols, ON–OFF–ON fluorescence, naked-eye recognition, smartphone-based RGB analysis, bioimaging

## Abstract

Copper ions (Cu^2+^) and intracellular biothiols are tightly coupled in cellular redox regulation, where copper–thiol coordination governs oxidative stress and metal homeostasis. However, analytical platforms capable of sequentially monitoring Cu^2+^ and biothiols within a single molecular system remain scarce. Herein, we report a coumarin-based fluorescent probe **XDP** that enables sequential ON–OFF–ON sensing of Cu^2+^ and biothiols through a coordination–competition mechanism. The imine (C=N) site of **XDP** selectively coordinates Cu^2+^, leading to fluorescence quenching arising from coordination-induced electronic perturbation and enhanced nonradiative decay. The probe exhibits a linear response toward Cu^2+^ over 1–80 μM with a detection limit of 0.108 μM. Subsequent competitive binding of biothiols (GSH, Cys, and Hcy) releases Cu^2+^ from the complex, thereby restoring fluorescence and enabling detection within 1–30 μM with submicromolar sensitivity. **XDP** also displays a large Stokes shift (135 nm), which minimizes spectral overlap and improves signal reliability. Notably, Cu^2+^ binding triggers a distinct color change that supports naked-eye detection and smartphone-based RGB quantification. The probe further enables visualization of Cu^2+^ and thiol-triggered signal recovery in living cells and zebrafish. This work establishes a versatile analytical platform for probing copper–thiol interactions in environmental and biological systems.

## 1. Introduction

Cu^2+^ participates in a wide range of essential biological processes through reversible redox cycling between Cu(I) and Cu(II), thereby modulating enzymatic activity and intracellular redox homeostasis [1,2,3]. Maintenance of copper homeostasis requires strict regulation of labile Cu^2+^, as excess free copper perturbs redox equilibrium and contributes to oxidative stress [4,5,6]. In cellular environments, copper speciation is closely associated with low-molecular-weight thiols such as cysteine (Cys), glutathione (GSH), and homocysteine (Hcy), which act as major intracellular reductants and readily coordinate with Cu^2+^ to form copper–thiol complexes that regulate metal availability [7,8,9]. However, the dynamic exchange between free Cu^2+^ and thiol-bound species renders their independent characterization challenging [5,10,11], thereby underscoring the need for analytical strategies capable of resolving sequential coordination and displacement processes.

Established techniques such as atomic absorption spectroscopy [12,13], inductively coupled plasma mass spectrometry [14,15], and electrochemical methods [16,17] provide accurate quantification of Cu^2+^ in environmental samples, yet remain unsuitable for real-time visualization in biological systems. Fluorescence-based approaches, while offering operational simplicity and high spatial resolution, have predominantly been developed for single-analyte detection [18,19]. Systems that enable stepwise monitoring of Cu^2+^ and thiols within a unified molecular framework remain scarce, primarily because such sequential sensing requires a well-defined coordination event with Cu^2+^ followed by a controlled thiol-mediated displacement process, while avoiding ambiguous or overlapping spectral responses [20,21,22,23].

Coumarin fluorophores have been extensively employed in probe design owing to their synthetic versatility and favorable photophysical properties [24,25]. Notably, coumarin derivatives often exhibit large Stokes shifts, which effectively minimize excitation–emission overlap and enhance signal discrimination in complex environments [26,27,28]. Nevertheless, most reported coumarin-based Cu^2+^ probes rely on single fluorescence response modes or involve multicomponent signaling pathways [29,30], whereas systems integrating visible colorimetric changes with portable readout capabilities remain relatively underexplored [31,32,33].

**Scheme 1:**
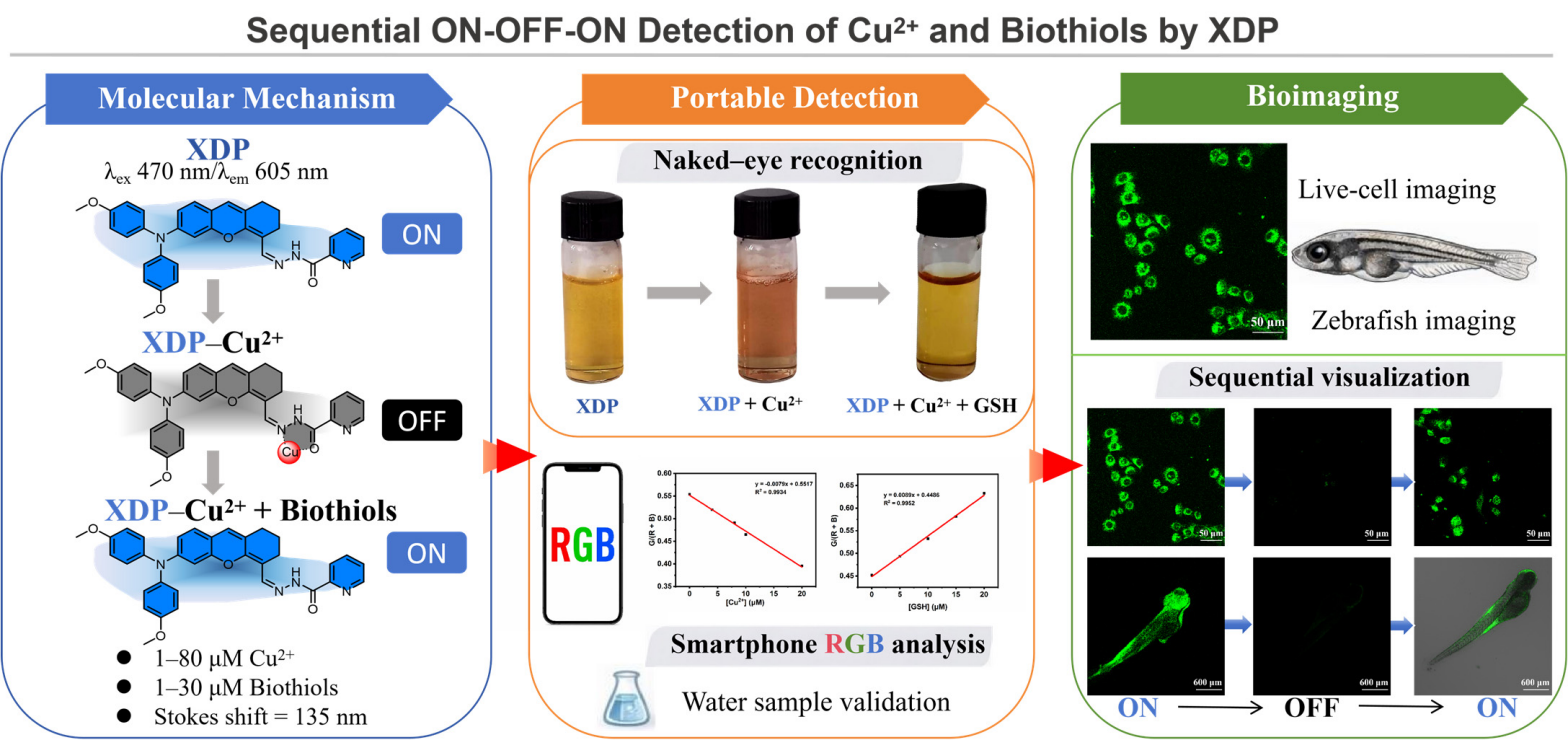
Sequential ON–OFF–ON fluorescence response of **XDP** toward Cu^2+^ and biothiols.

Herein, we report a coumarin-based fluorescent probe (**XDP**) that exhibits a sequential ON–OFF–ON fluorescence response toward Cu^2+^ and biothiols. As illustrated in Scheme 1, the imine (C=N) moiety in **XDP** serves as a coordination site for Cu^2+^, leading to fluorescence quenching, while subsequent competitive interaction with thiols restores emission, allowing stepwise detection within a single molecular framework. **XDP** exhibits a large Stokes shift of 135 nm, which reduces spectral overlap and improves signal discrimination in aqueous and biological environments. In addition to fluorescence switching, Cu^2+^ binding is accompanied by a distinct visible color change, enabling direct visual recognition and smartphone-assisted RGB analysis. The analytical applicability of the system was validated in environmental water samples and further demonstrated through fluorescence imaging in living cells and zebrafish.

## 2. Experimental

### 2.1. Instruments and Reagents

Except for those used in cell experiments, all chemicals and reagents were of analytical grade and used as received without further purification. The CCK-8 assay kit was obtained from Beijing Solarbio Science & Technology Co, Ltd. (Beijing, China). Ultrapure water (18.25 MΩ·cm, Millipore-Q) was used for the preparation of all aqueous solutions. For cell experiments, PBS and DMSO were of cell culture grade. Nuclear magnetic resonance (NMR) spectra were recorded on a Bruker AVANCE II 600 MHz spectrometer (Rheinstetten, Germany), with tetramethylsilane (TMS) employed as the internal standard. High-resolution mass spectrometry (HRMS) analyses were carried out using a Thermo Fisher Q Exactive UHMR Quadrupole-Orbitrap mass spectrometer (Bremen, Germany) operated in ESI/APCI modes. Fluorescence measurements were performed on an F97 fluorescence spectrophotometer (Shanghai, China). Confocal fluorescence imaging of cells was conducted using an Olympus FV3000 confocal laser scanning microscope (Tokyo, Japan). Ultraviolet (UV) measurements were acquired on a Jinghua UV2700 ultraviolet spectrophotometer (Shanghai, China).

### 2.2. Synthesis of Probe **XDP**

The synthesis of probe **XDP** was carried out according to the following procedure (Scheme S1). Synthesis of Compound 1. CHCl_3_ (50.0 mL) and DMF (12.0 mL, 155.0 mmol) were mixed and cooled to 0 °C in an ice-water bath with stirring. PBr_3_ (9.0 mL, 95.8 mmol) was then added dropwise while maintaining the reaction temperature below 0 °C, and the mixture was stirred for an additional 1 h. Subsequently, cyclohexanone (4.0 mL, 38.6 mmol) was slowly introduced into the reaction mixture via syringe. The ice bath was removed, and the reaction was allowed to proceed at room temperature for 18 h. After completion, the reaction mixture was slowly poured into ice water and neutralized to pH = 7 by the gradual addition of solid NaHCO_3_. The organic phase was extracted with dichloromethane, washed twice with saturated brine, dried over anhydrous Na_2_SO_4_, filtered, and concentrated under reduced pressure to afford a yellow oily product [34].

Synthesis of Compound 2. Under a nitrogen atmosphere, compound 1 (520.0 mg, 2.75 mmol, 1.0 equiv) and Cs_2_CO_3_ (2.69 g, 3.0 equiv) were dissolved in anhydrous DMF (20.0 mL). A solution of 4-bromo-2-hydroxybenzaldehyde (1.10 g, 2.0 equiv) in DMF (2 mL) was then added dropwise to the reaction mixture at room temperature. The resulting mixture was stirred for 48 h until TLC analysis indicated completion of the reaction. The mixture was subsequently filtered to remove inorganic salts, and the filtrate was washed twice with deionized water and extracted with ethyl acetate. The combined organic layers were dried over anhydrous Na_2_SO_4_ and concentrated under reduced pressure. The crude product was purified by silica gel column chromatography (PE/EA = 20:1, *v*/*v*) to afford compound 2 as an orange-yellow solid (296.4 mg, 36.7%) [34].

Synthesis of Compound 3. Under a nitrogen atmosphere, compound 2 (250.0 mg, 0.86 mmol, 1.0 equiv), 4,4-dimethoxydiphenylamine (197.2 mg, 1.0 equiv), Pd_2_(dba)_3_ (11.8 mg, 1.3 mmol%), DavePhos (5.0 mg, 1.3 mmol%), and Cs_2_CO_3_ (700.5 mg, 2.5 equiv) were dissolved in 1,4-dioxane (30.0 mL). The reaction mixture was stirred at 95 °C for 18 h until TLC indicated complete consumption of the starting material. After cooling to room temperature, the mixture was filtered to remove solid residues. The filtrate was washed twice with water and extracted with ethyl acetate. The combined organic layers were dried over anhydrous Na_2_SO_4_, filtered, and concentrated under reduced pressure. The residue was purified by silica gel column chromatography (PE/EA = 10:1, *v*/*v*) to yield compound 3 as an orange solid (183.0 mg, 48%) [35]. 1H NMR (600 MHz, CDCl3) δ 10.18 (s, 1H), 7.11–7.07 (m, 4H), 6.92 (d, J = 8.4 Hz, 1H), 6.89–6.85 (m, 4H), 6.60 (s, 1H), 6.59–6.54 (m, 2H), 3.81 (s, 6H), 2.54 (t, J = 5.5 Hz, 2H), 2.42 (t, J = 6.0 Hz, 2H), 1.70 (q, J = 6.1 Hz, 2H). 13C NMR (151 MHz, CDCl3) δ 187.43, 161.10, 156.75, 153.22, 150.75, 139.52, 127.45, 127.12, 126.94, 125.52, 114.92, 114.55, 113.78, 112.07, 104.71, 55.47, 29.94, 21.53, 20.53.

Synthesis of probe **XDP**. Under a nitrogen atmosphere, compound 3 (100.0 mg, 0.23 mmol, 1.0 equiv) and 2-pyridinecarbohydrazide (31.5 mg, 1.0 equiv) were dissolved in anhydrous ethanol, followed by the addition of one drop of glacial acetic acid as a catalyst. The reaction mixture was refluxed for 4 h, and the progress was monitored by TLC. Upon completion, the solvent was removed under reduced pressure. The residue was washed with water and extracted with ethyl acetate. The organic layer was dried over anhydrous Na_2_SO_4_ and concentrated under reduced pressure. The crude product was purified by column chromatography to afford probe **XDP** as a dark orange solid (67.1 mg, 52.2%). 1H NMR (600 MHz, CDCl3) δ 10.77 (s, 1H), 8.57 (dt, J = 4.6, 1.3 Hz, 1H), 8.47 (s, 1H), 8.29 (dt, J = 7.9, 1.1 Hz, 1H), 7.87 (td, J = 7.7, 1.7 Hz, 1H), 7.45 (ddd, J = 7.6, 4.7, 1.2 Hz, 1H), 7.09 (d, J = 8.9 Hz, 4H), 6.88 (d, J = 8.9 Hz, 4H), 6.81 (d, J = 8.3 Hz, 1H), 6.53 (d, J = 2.2 Hz, 1H), 6.49 (dd, J = 8.3, 2.3 Hz, 1H), 6.29 (s, 1H), 3.82 (s, 6H), 2.70 (t, J = 6.0 Hz, 2H), 2.50 (t, J = 6.1 Hz, 2H), 1.76–1.71 (m, 1H). 13C NMR (151 MHz, CDCl3) δ 159.33, 156.34, 153.49, 152.47, 149.82, 149.62, 147.92, 146.07, 140.08, 137.48, 127.19, 126.98, 126.33, 126.28, 122.73, 122.50, 114.80, 114.64, 114.37, 108.11, 105.36, 55.46, 29.98, 23.63, 20.77. HRMS-ESI (*m*/*z*) for C34H31N4O4+: calcd 559.2340, found 559.2345.

### 2.3. Fluorometric Detection of Cu^2+^ and Biothiols

For Cu^2+^ sensing, a series of test solutions were prepared by introducing varying aliquots of a Cu^2+^ stock solution (1 mM) into a probe **XDP** solution (0.05 mM, 400 μL), followed by dilution with Tris-HCl buffer (10 mM, pH 7.4) to a total volume of 2.0 mL. For sequential biothiol detection, 20 μL of a Cu^2+^ stock solution (1 mM) was first added to the probe **XDP** solution (0.05 mM, 400 μL) and allowed to equilibrate at room temperature for 5 min. Subsequently, appropriate amounts of biothiol stock solutions (1 mM) and Tris-HCl were introduced to afford a final assay volume of 2.0 mL prior to fluorescence measurements. Fluorescence emission spectra were recorded in the absence and presence of analytes using an excitation wavelength of 470 nm, with emission collected at 605 nm. The emission slit width was maintained at 10.0 nm throughout all measurements.

### 2.4. Real Samples Treatment

Water samples were collected from the campus lake and tap water of Hubei University of Science and Technology. All samples were filtered through a 0.22 μm membrane to remove insoluble particulates and then analyzed according to the standard procedure described above. Urine and serum samples were obtained from the Second Affiliated Hospital of Hubei University of Science and Technology, anonymized before analysis, and used only for method validation. For urine analysis, normal adult urine was diluted to 10% and analyzed using the same procedure. For serum samples, 1 mL of acetonitrile was added to 1 mL of serum to reduce interference from the high-protein matrix. After stirring for 5 min, the mixture was centrifuged at 10,000 rpm for 15 min. The supernatant was collected, filtered through a 0.22 μm microporous membrane, diluted 100-fold, and then analyzed according to the above-mentioned method. Each experiment was independently repeated three times, and the average results were reported.

### 2.5. In Vitro Cytotoxicity Evaluation

Cellular viability was evaluated using a CCK-8 assay. Cells were seeded into 96-well plates and allowed to adhere, followed by treatment with probe **XDP** at varying concentrations. After incubation for 24 h, CCK-8 solution was added to each well and the plates were further incubated for 2 h. Cell viability was subsequently quantified by recording the absorbance at 450 nm using a microplate reader. Each experiment was independently repeated three times.

### 2.6. Cellular Imaging Experiments

HeLa cells were seeded in confocal culture dishes and incubated under standard culture conditions for 24 h to allow sufficient cell adhesion. The cells were then randomly divided into different experimental groups to systematically investigate the sequential fluorescence response of probe **XDP** toward Cu^2+^ and biothiols in living cells. All experiments were conducted with three independent repetitions, and the results were averaged to reduce randomness.

To evaluate the intracellular imaging performance of **XDP** toward Cu^2+^ and biothiols, five experimental groups were established: NEM + **XDP** (a), NEM + **XDP** + Cu^2+^ (b), NEM + **XDP–Cu^2+^** complex (c), **XDP–Cu^2+^** complex (d), and NEM + **XDP–Cu^2+^** complex + GSH (e). For group (a), cells were pretreated with N-ethylmaleimide (NEM, 200 μM) for 0.5 h to deplete endogenous biothiols prior to incubation with **XDP** (10 μM) for 1 h. Subsequently, Cu^2+^ (30 μM) was introduced into the NEM-pretreated cells that had been incubated with **XDP**, followed by an additional 1 h of incubation forming group (b). This group was designed to assess the fluorescence response of **XDP** toward Cu^2+^ under thiol-depleted conditions. For group (c), cells were first pretreated with NEM for 0.5 h. **XDP** was pre-coordinated with Cu^2+^ to form the **XDP–Cu^2+^** complex (10 μM), which was then added to the NEM-treated cells followed by an additional 1 h of incubation. This group served as a blank control for thiol recognition. In group (d), cells without NEM pretreatment were incubated with the **XDP–Cu^2+^** complex for 1 h to evaluate the response toward endogenous biothiols. In addition, based on group (c), exogenous GSH (50 μM) was added and incubated for 1 h to establish group (e), allowing assessment of the detection capability toward exogenous biothiols. After all treatments, the cells were gently washed three times with PBS to remove excess probe and unbound species. Fluorescence imaging was performed using a laser scanning confocal microscope (LSCM) under identical imaging parameters for all groups.

### 2.7. Imaging in Zebrafish

Zebrafish embryos were obtained from Shanghai Feixi Biotechnology Co., Ltd. (Shanghai, China) and maintained in embryo medium under standard conditions at 28 °C. Three-day-post-fertilization (3 dpf) larvae were randomly divided into five groups. All experiments were conducted with three independent repetitions. Group (a): Larvae were pretreated with N-ethylmaleimide (NEM, 50 μM) for 0.5 h to deplete endogenous biothiols, followed by incubation with **XDP** (10 μM) for 1 h. Group (b): Based on group (a), Cu^2+^ was subsequently introduced into larvae pretreated with NEM and incubated with **XDP** for 1 h to evaluate the fluorescence response of the **XDP** toward Cu^2+^ under thiol-depleted conditions. Group (c): Larvae were first pretreated with NEM (50 μM) for 0.5 h. **XDP** was pre-coordinated with Cu^2+^ to form the **XDP–Cu^2+^**  complex (10 μM) for 1 h, which was then added to the NEM-treated larvae. This group served as a blank control for thiol recognition. Group (d): Larvae without NEM pretreatment were incubated with the **XDP–Cu^2+^** complex for 1 h to assess the response toward endogenous biothiols. Group (e): On the basis of group (c), exogenous GSH (50 μM) was added and incubated for 1 h to evaluate the capability of the system to detect exogenous biothiols. After the respective treatments, all larvae were washed three times with embryo medium and fixed with 4% paraformaldehyde. The fixed larvae were transferred to confocal imaging dishes and fluorescence images were acquired using a laser scanning confocal microscope (LSCM) under identical imaging parameters.

## 3. Results

### 3.1. Synthesis and Spectral Response of Probe XDP

Although numerous fluorescent probes have been developed for the individual detection of Cu^2+^ or biothiols, systems that integrate naked-eye visualization, smartphone-assisted readout, and sequential sensing detection within a single molecular platform remain relatively underexplored. In light of these considerations, a coumarin scaffold was chosen as the fluorophore, while diphenylamine was introduced as an electron-donating unit to construct a D–π–A conjugated architecture. Such a configuration leads to red-shifted emission accompanied by a large Stokes shift, which improves signal discrimination and suppresses background interference. Meanwhile, pyridine hydrazide was incorporated as a Cu^2+^-binding unit to establish a coordination–competition-responsive system. On this basis, the fluorescent probe **XDP** was rationally designed and synthesized, allowing portable detection through direct visual observation in combination with smartphone-assisted analysis.

**Figure 1:**
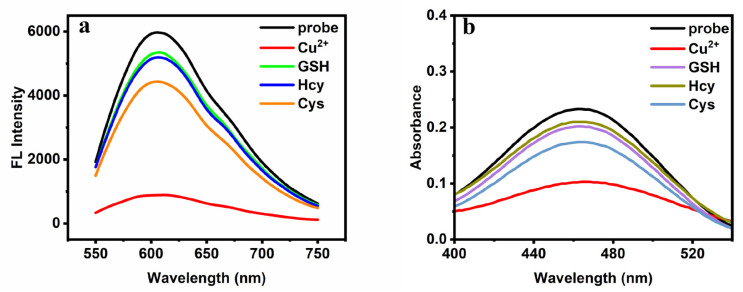
(**a**) Fluorescence emission spectra of **XDP** (10 μM) + Cu^2+^ (10 μM) and **XDP–Cu^2+^** (10 μM) + biothiols (30 μM) in DMSO/Tris-HCl buffer (1:1, *v*/*v*, 10 mM, pH 7.4). λex = 470 nm; (**b**) Absorption spectra of **XDP** + Cu^2+^ (10 μM) and **XDP–Cu^2+^** (10 μM) + biothiols (30 μM) in DMSO/Tris-HCl buffer (1:1, *v*/*v*, 10 mM, pH 7.4).

As shown in Figures S1–S4, the structure of **XDP** was confirmed by NMR, verifying the successful construction of the target molecule. To evaluate its spectroscopic response, UV–vis absorption and fluorescence measurements were carried out in the absence and presence of Cu^2+^ and biothiols. As illustrated in Figure 1, free **XDP** displays a maximum absorption at 466 nm along with strong fluorescence emission at 605 nm (λex = 470 nm), which is characteristic of an intramolecular charge transfer (ICT) process. The resulting Stokes shift of 135 nm minimizes spectral overlap between excitation and emission, thereby improving signal discrimination. Upon introduction of Cu^2+^, pronounced fluorescence quenching is observed, which can be attributed to coordination-induced electronic perturbation, metal-involved charge transfer, and paramagnetic effects. Subsequent introduction of biothiols leads to competitive binding with Cu^2+^, shifting the coordination equilibrium and restoring both absorption and emission signals. This reversible process gives rise to a characteristic “ON–OFF–ON” fluorescence response, allowing stepwise detection of Cu^2+^ and biothiols within a single system.

### 3.2. Optimization of Sensing Conditions

In practical applications, response time, pH, and temperature are critical parameters for evaluating sensing performance. Accordingly, the fluorescence responses of **XDP** toward Cu^2+^ and biothiols were systematically optimized under varied conditions. As shown in Figure S5, upon exposure to Cu^2+^, the fluorescence intensity of **XDP** at 605 nm decreases rapidly with incubation time and reaches a plateau within 2 min (Figure S5a), reflecting fast coordination kinetics between the imine recognition unit and Cu^2+^, which is advantageous for rapid detection. Following the addition of GSH, the fluorescence signal gradually recovers and stabilizes within approximately 15 min (Figure S5d), indicating efficient competitive binding of biothiols to Cu^2+^ and supporting the sequential sensing process.

The effect of pH on sensing behavior was further investigated (Figure S5b,e). **XDP** exhibits stable and pronounced responses toward both Cu^2+^ and biothiols within the pH range of 6.0–7.4. Under acidic conditions, protonation of the imine nitrogen reduces the availability of its lone pair electrons for coordination, thereby weakening Cu^2+^ binding [36]. In contrast, under alkaline conditions, the increased concentration of hydroxide ions promotes the formation of copper hydroxide complexes or precipitates, decreasing the effective concentration of free Cu^2+^ available for coordination [37]. For biothiols, the thiol group predominantly exists in the protonated form (–SH) in acidic media, resulting in weaker coordination to Cu^2+^ and diminished displacement of the **XDP–Cu^2+^** complex. At neutral to slightly alkaline pH, deprotonation of the thiol group enhances its coordination ability, thereby promoting competitive binding [38]. Therefore, physiological pH (7.4) was selected for subsequent studies.

The influence of incubation temperature on sensing performance was also examined. As shown in Figure S5c, fluorescence quenching of **XDP** by Cu^2+^ is most pronounced at 37 °C, whereas lower temperatures may limit molecular collisions and coordination kinetics, and higher temperatures can reduce the stability of the **XDP–Cu^2+^** complex. Notably, this optimal temperature is close to physiological temperature, supporting the applicability of the probe in biological imaging. In contrast, thiol-triggered fluorescence recovery process shows minimal temperature dependence (Figure S5f). According to the hard and soft acids and bases (HSAB) theory, the thiol group (soft base) exhibits a favorable coordination tendency toward Cu^2+^ (borderline acid), providing a thermodynamic driving force for competitive binding and fluorescence restoration. Consequently, the sequential sensing system maintains reliable performance within a moderate temperature range.

### 3.3. Analytical Performance of XDP

**Figure 2:**
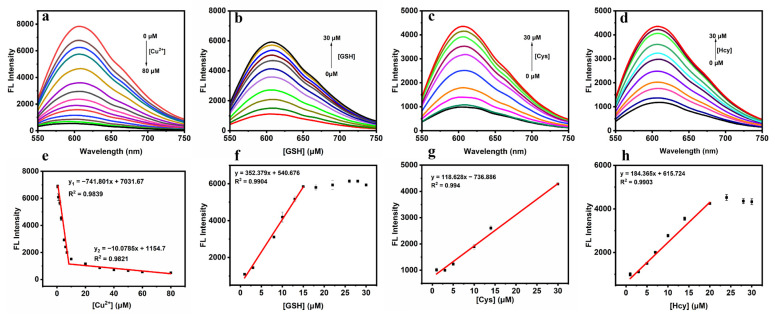
Results are averaged over three independent runs. (**a**–**d**) Fluorescence response of **XDP** (10 μM) to Cu^2+^ and **XDP–Cu^2+^** to biothiols (GSH/Cys/Hcy); (**e**–**h**) Linear correlations between fluorescence intensity and Cu^2+^/biothiol levels.

To assess the quantitative sensing performance of **XDP** toward Cu^2+^ and biothiols, concentration-dependent fluorescence titration were conducted under the optimized conditions. As shown in Figure 2a,e, the fluorescence intensity of **XDP** at 605 nm decreases progressively with increasing Cu^2+^ concentration over the range of 0–80 μM, exhibiting a well-defined concentration-dependent response. A good linear relationship is observed within 1–80 μM, affording a detection limit of 0.108 μM, which supports the reliable quantification of Cu^2+^. After formation of the **XDP–Cu^2+^** complex (10 μM), the fluorescence recovery induced by biothiols was further examined. Taking GSH as an example, the fluorescence intensity increases gradually with increasing GSH concentration (0–30 μM), and a linear relationship is obtained within 1–15 μM (Figure 2b,f), with a detection limit of 0.116 μM. Similar concentration-dependent fluorescence enhancement is observed for Cys (Figure 2c,g) and Hcy (Figure 2d,h), with detection limits of 0.342 μM and 0.233 μM, respectively. These results demonstrate that **XDP** affords consistent and reproducible responses toward multiple biothiols. Notably, the Cu^2+^-induced fluorescence quenching and the subsequent thiol-triggered fluorescence restoration correspond to the “OFF” and “ON” stages, respectively, allowing sequential quantitative detection within a single molecular platform. The clear concentration dependence and linear response profiles establish a quantitative foundation for applications in complex environmental and biological matrices. In addition, the photostability of **XDP** was evaluated under continuous excitation. As shown in Figure S6, the fluorescence intensity at 605 nm remains essentially unchanged over 30 min of irradiation, indicating good resistance to photobleaching.

Compared with previously reported fluorescent probes for Cu^2+^ and biothiol detection (Table S1), **XDP** shows favorable overall performance in terms of detection limits, linear dynamic ranges, and readout modes. A key feature of **XDP** is that it enables a sequential fluorescence response to Cu^2+^ binding and biothiol-mediated competitive displacement within a single molecular platform, which can be further used for quantitative analysis. Meanwhile, the Cu^2+^-induced visible color change allows naked-eye recognition of the Cu^2+^/biothiol response process, while smartphone-based RGB analysis converts the color variation into quantifiable digital signals, thereby extending the potential of this system for portable on-site detection.

### 3.4. Selectivity

**Figure 3:**
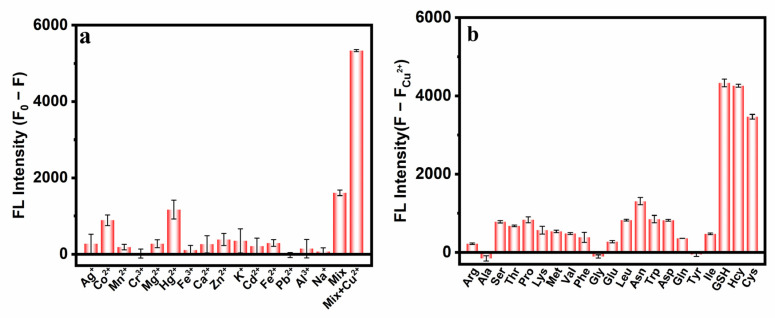
Results are averaged over three independent runs. (**a**) Selectivity and interference evaluation of **XDP** (10 μM) toward various metal ions; (**b**) Fluorescence response of the **XDP–Cu^2+^** complex (10 μM) toward different amino acids. Cu^2+^, biothiols, and other interferents were used at 10, 30, and 50 μM, respectively.

To evaluate the performance of **XDP** in complex environments, its selectivity toward Cu^2+^ and biothiols was systematically investigated. As shown in Figure 3a, various competing metal ions produced negligible fluorescence changes at 605 nm, whereas Cu^2+^ induced pronounced quenching, indicating a highly selective response of **XDP** toward Cu^2+^. The observed selectivity can be attributed to the structural features of **XDP**, in which the imine (C=N) and pyridine hydrazide units provide N-donor sites suitable for metal coordination, while Cu^2+^ exhibits favorable binding geometry and affinity under the present conditions. By comparison, alkali and alkaline earth metal ions show weak interactions with these donor sites, whereas other transition metal ions fail to induce sufficient electronic perturbation to trigger fluorescence quenching. Notably, the fluorescence response toward Cu^2+^ remains essentially unchanged in the presence of competing ions, suggesting that interference from these species does not significantly disrupt the **XDP–Cu^2+^** interaction.

The selectivity toward biothiols was further assessed using the **XDP–Cu^2+^** complex (Figure 3b). Significant fluorescence recovery was observed only for thiol-containing species (GSH, Cys, and Hcy), whereas other amino acids produced negligible effects. This behavior arises from the stronger affinity of sulfur donors for Cu^2+^ relative to oxygen- or nitrogen-based groups, which shifts the coordination equilibrium and releases **XDP** from the metal-bound state, thereby restoring fluorescence. Collectively, the selective fluorescence switching behavior of **XDP** toward Cu^2+^ and thiol-containing biomolecules underscores its suitability for use in complex chemical and biological environments.

### 3.5. Smartphone-Assisted Visual Detection

**Figure 4:**
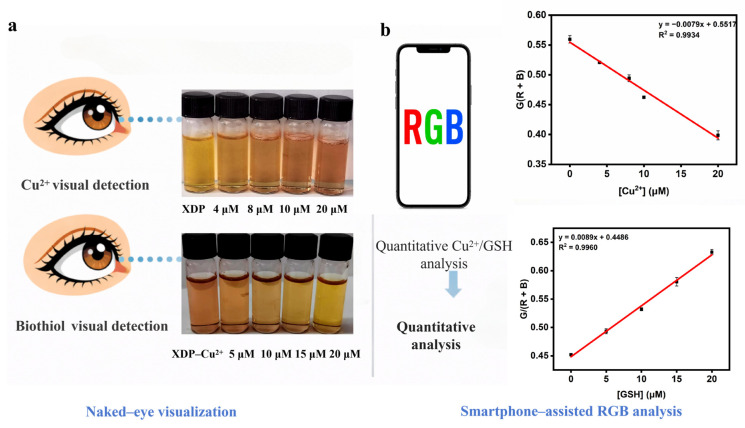
(**a**) Naked-eye visualization of the sequential response of **XDP** to Cu^2+^ and GSH; (**b**) Smartphone-assisted RGB analysis of **XDP** toward Cu^2+^ and the **XDP–Cu^2+^** complex toward GSH.

To facilitate portable and on-site analysis, a smartphone-assisted visual–fluorescence dual-mode detection strategy was developed. Upon addition of Cu^2+^, the **XDP** solution undergoes a distinct color change under ambient light (Figure 4a), which can be directly recognized by the naked eye without UV illumination. This response originates from coordination-induced modulation of the electronic structure, which alters the absorption profile and is accompanied by fluorescence quenching, thereby translating molecular-level interactions into a perceptible macroscopic signal. Building on this visual response, images of the solution were captured using a smartphone, and the corresponding RGB parameters were extracted through digital analysis. All RGB measurements were performed under ambient light conditions. During the measurements, all samples were placed on a white background and photographed using the rear camera of the same smartphone model at a fixed distance. To minimize variations in color acquisition caused by camera settings, automatic exposure and automatic white balance were disabled, and high-resolution images of the solution samples were collected. The RGB values were then extracted from the images using a smartphone application, and G/(R + B) was used as the color intensity parameter for analysis. To reduce random experimental errors, each sample was measured in triplicate. As the Cu^2+^ concentration increases, systematic variations in RGB values are observed, which correlate with the gradual color change of the solution (Figure 4b). Quantitative fitting of these parameters against Cu^2+^ concentration yields a satisfactory linear relationship, indicating that the visual signal can be reliably digitized for quantitative analysis.

As presented in Figure 4, formation of the **XDP–Cu^2+^** complex was followed by gradual recovery of the solution color upon addition of biothiols, accompanied by corresponding reverse changes in RGB values. Specifically, the G/(R + B) value decreased linearly with increasing Cu^2+^ concentration, whereas in the **XDP–Cu^2+^** system, it increased progressively with increasing GSH concentration, indicating that smartphone-derived RGB signals can reflect both Cu^2+^ binding and GSH-mediated competitive displacement. Further statistical analysis showed clear differences among different concentration groups with small variations (Figure S7a,b), consistent with the fluorescence spectroscopic results and supporting the repeatability and concentration-resolving capability of this digital readout. Compared with conventional spectroscopic, which requires dedicated instrumentation, smartphone-based RGB analysis converts visible color changes into quantifiable signals and provides a simpler and more accessible readout for rapid on-site screening and semi-quantitative detection under resource-limited conditions, thereby broadening the potential of **XDP** for portable analysis.

### 3.6. Determination of Cu^2+^ in Real Water Samples

To evaluate the applicability of **XDP** in real samples, spiked recovery experiments for Cu^2+^ were performed in lake water, tap water, urine, and serum samples using both fluorescence and smartphone-based RGB methods. As summarized in Table 1, the recoveries obtained in lake water and tap water at different spiking levels ranged from 96.0% to 119.0%, with relative standard deviations (RSDs) of 1.06–4.82%, indicating good accuracy and repeatability in real water matrices. Trace Cu^2+^ signals were detected in the unspiked water samples, which may be attributed to environmental background levels or naturally occurring trace metal ions in aquatic systems. To further examine the applicability of this method in complex biological matrices, spiked recovery experiments were also conducted in urine and serum samples. The recoveries in urine samples ranged from 100.5% to 108.0%, with RSDs of 2.39–4.18%, while those in serum samples ranged from 100.1% to 111.0%, with RSDs of 1.76–3.53%. These results suggest that, after appropriate dilution or protein precipitation, the urine and serum matrices had limited influence on the Cu^2+^ detection performance of **XDP**. Notably, the fluorescence responses observed in spiked samples were comparable to those in buffer systems, suggesting that matrix components exert negligible interference on the coordination behavior of the **XDP–Cu^2+^** system. Consistent concentration-dependent variations were also obtained from smartphone-based RGB analysis, which correlate well with the fluorescence results, thereby supporting the reliability of the visual–digital dual-mode detection strategy in practical applications.

### 3.7. Response Mechanism

To elucidate the recognition mechanism of **XDP** toward Cu^2+^, the binding stoichiometry, complex composition, and electronic structural changes were systematically investigated. As indicated by the Job’s plot (Figure S7c), the maximum fluorescence change occurs at a molar fraction of approximately 0.5, supporting a 1:1 binding ratio between **XDP** and Cu^2+^. This binding mode is further supported by ESI-MS analysis (Figure S8). Free **XDP** shows a peak at *m*/*z* 559.7 corresponding to [C_34_H_30_N_4_O_4_ + H]^+^, while a new signal at *m*/*z* 658.8 appears upon Cu^2+^ addition, which is assigned to [C_34_H_30_N_4_O_4_ + Cu^2+^ + 2H_2_O]^+^. These results indicate the formation of an **XDP–Cu^2+^** complex. Based on the molecular structure of **XDP** and related Cu^2+^-responsive Schiff base/pyridine-containing probes, the imine and pyridine hydrazide moieties are proposed as the probable coordination sites through their accessible N/O donor atoms.

**Figure 5:**
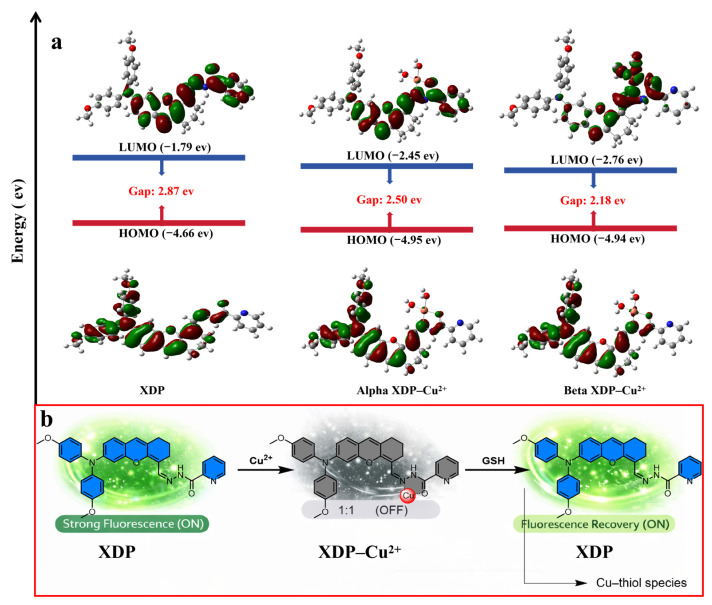
(**a**) Frontier molecular orbital (HOMO and LUMO) distributions of **XDP** and its Cu^2+^ complex obtained from density functional theory (DFT) calculations; (**b**) Schematic illustration of the sequential sensing mechanism of **XDP** toward Cu^2+^ and biothiols.

To gain insight into the electronic origin of fluorescence modulation, density functional theory (DFT) calculations were performed (Figure 5). For free **XDP**, the HOMO is primarily localized on the diphenylamine donor unit, whereas the LUMO is distributed over the coumarin acceptor framework, which is characteristic of a D–π–A system and supports an ICT transition. The calculated HOMO–LUMO energy gap of the free probe is 2.87 eV. After coordination with Cu^2+^, the frontier orbital distribution is significantly altered, with increased electron density around the coordination site, indicating involvement of the metal center in the electronic structure. Correspondingly, the energy gaps decrease to 2.50 eV (α) and 2.18 eV (β), suggesting that Cu^2+^ coordination perturbs the original electronic structure of **XDP**. Such coordination-induced electronic modulation, together with the paramagnetic nature of Cu^2+^, may weaken the ICT-related emission and facilitate nonradiative deactivation, leading to fluorescence quenching. Upon subsequent addition of biothiols, competitive coordination with Cu^2+^ disrupts the **XDP–Cu^2+^** complex, releasing the probe and restoring the ICT transition and fluorescence emission. Collectively, the combined stoichiometric, spectrometric, and theoretical results support a coordination–competition mechanism that accounts for the sequential “ON–OFF–ON” fluorescence response.

### 3.8. Cell Imaging

Encouraged by the solution-phase results, the applicability of **XDP** for detecting Cu^2+^ and biothiols in living cells was further investigated. Prior to imaging, cytocompatibility was assessed by a CCK-8 assay, which showed that cell viability remained above 85% after 24 h incubation across the tested concentration range (Figure S9), indicating acceptable biocompatibility under the experimental conditions.

**Figure 6:**
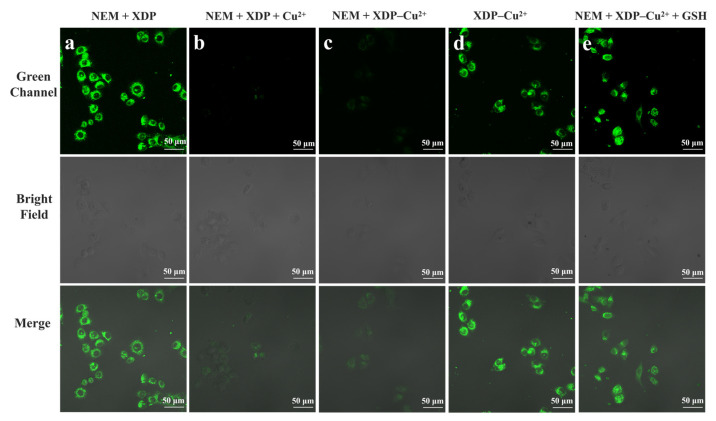
HeLa cells confocal imaging: (**a**) **XDP** + 200 μM NEM; (**b**) **XDP** + NEM + 30 μM Cu^2+^; (**c**) **XDP–Cu^2+^** + NEM; (**d**) **XDP–Cu^2+^**; (**e**) **XDP–Cu^2+^** + NEM + 50 μm GSH. λex = 488 nm, λem = 500–600 nm.

Fluorescence imaging was then performed following the established treatment protocol. As shown in Figure 6a, cells pretreated with NEM to deplete endogenous thiols exhibited bright intracellular fluorescence upon incubation with **XDP**, suggesting efficient cellular uptake and retention of the emissive state in the absence of metal ions. Upon addition of Cu^2+^ under thiol-depleted conditions (Figure 6b), the fluorescence intensity decreased markedly, leaving only weak background signals, which is consistent with the quenching behavior observed in solution and indicates that **XDP** retains its responsiveness toward Cu^2+^ in the cellular environment. Cells treated with the preformed **XDP–Cu^2+^** complex after NEM pretreatment (Figure 6c) displayed persistently low fluorescence, consistent with a quenched state. In contrast, when NEM pretreatment was omitted (Figure 6d), fluorescence was restored after incubation with the **XDP–Cu^2+^** complex, indicating that endogenous thiols competitively interact with Cu^2+^ and disrupt the coordination equilibrium, thereby releasing the probe. A similar recovery was observed upon addition of exogenous GSH (Figure 6e), further confirming the thiol-responsiveness of the system in the intracellular environment. Quantitative analysis of the average fluorescence intensity in HeLa cells (Figure S10a) is consistent with the imaging results, confirming that **XDP** can follow the Cu^2+^-induced quenching and thiol-triggered fluorescence recovery process under cellular conditions. The fluorescence variations observed across different treatment groups follow the same “ON–OFF–ON” sequence as that identified in solution, indicating that the coordination–competition mechanism remains operative under cellular conditions and supports stepwise detection of Cu^2+^ and biothiols in a physiological context.

### 3.9. In Vivo Imaging in Zebrafish

**Figure 7:**
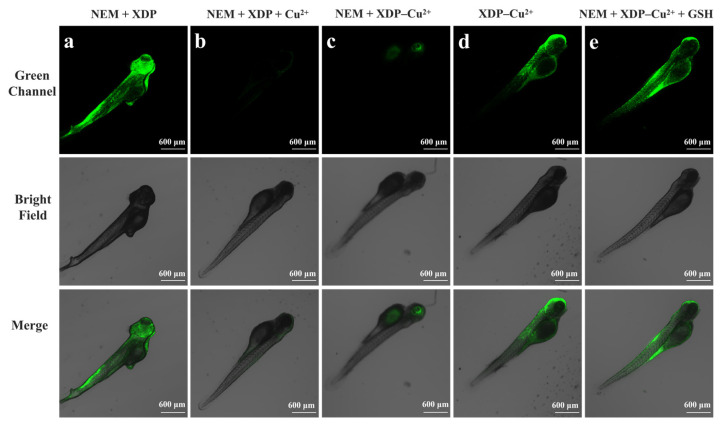
Zebrafish confocal imaging: (**a**) **XDP** + 50 μm NEM; (**b**) **XDP** + NEM + 30 μm Cu^2+^; (**c**) **XDP–Cu^2+^** + NEM; (**d**) **XDP–Cu^2+^**; (**e**) **XDP–Cu^2+^** + NEM + 50 μm GSH. λex = 488 nm, λem = 500–600 nm.

To further evaluate the performance of **XDP** in a whole-organism context, fluorescence imaging was conducted using 3 dpf zebrafish larvae. As shown in Figure 7a, larvae pretreated with NEM and subsequently incubated with **XDP** displayed pronounced fluorescence, indicating that the probe remains emissive under physiological conditions. Upon introduction of Cu^2+^ under the same thiol-depleted background (Figure 7b), fluorescence was significantly attenuated, reflecting coordination-induced quenching in vivo. Larvae pretreated with NEM and exposed to the preformed **XDP–Cu^2+^** complex (Figure 7c) maintained low fluorescence intensity, consistent with a stable quenched state in the absence of competing thiols. In contrast, when NEM pretreatment was omitted (Figure 7d), fluorescence recovery was observed after incubation with the **XDP–Cu^2+^** complex, which can be attributed to competitive coordination of endogenous biothiols that shifts the binding equilibrium and releases the probe. A comparable enhancement was obtained upon addition of exogenous GSH under thiol-depleted conditions (Figure 7e), further confirming the thiol-responsiveness of the system in vivo. The quantitative analysis of average fluorescence intensity in zebrafish is consistent with the imaging results, as shown in Figure S10b. The fluorescence changes observed across different treatment groups, which follow a stepwise modulation pattern, indicate that the coordination–competition equilibrium between **XDP** and Cu^2+^ remains operative in the physiological environment of zebrafish, thereby enabling visualization of copper–thiol interactions at the organismal level.

## 4. Conclusions

In summary, a coordination–competition-driven sequential sensing strategy is established for staged recognition of Cu^2+^ and biothiols within a single molecular framework. By integrating a pyridine hydrazide unit into a D–π–A coumarin scaffold, **XDP** operates through a reversible metal-binding and thiol-displacement process, in which Cu^2+^-induced quenching is followed by thiol-triggered fluorescence recovery. Stoichiometric analysis, mass spectrometry, and DFT calculations consistently support a 1:1 coordination mode and define the electronic basis of the switching behavior. Distinct from conventional single-analyte probes, this system enables stepwise differentiation of metal binding and thiol-mediated displacement within a unified platform, thereby resolving the coupled nature of copper–thiol interactions. **XDP** exhibits reliable concentration-dependent responses with rapid kinetics and high selectivity, while a large Stokes shift (135 nm) ensures effective signal discrimination in complex environments. The combination of spectroscopic response, direct visual readout, and smartphone-based RGB analysis further extends detection from instrumental measurement to portable and on-site formats. The applicability of the strategy is validated across environmental samples and biological systems, where the coordination–competition equilibrium remains operative from solution to cellular and organismal levels. This work defines a generalizable design principle for sequential fluorescence sensing and provides a practical route for interrogating dynamically coupled metal–thiol systems.

## Figures and Tables

**Table 1:** Determination of Cu^2+^ in real samples.

Sample	Method	Found (μM)	Added (μM)	Detected (μM)	Recovery (%)	RSD (%)
Lake Water	Fluorescence	0.21 ± 0.04	2	2.59 ± 0.03	119.0	1.16
			20	20.9 ± 1.00	103.5	4.78
	RGB	0.28 ± 0.06	2	2.52 ± 0.09	112.0	3.57
			20	20.88 ± 0.59	103.0	2.83
Tap Water	Fluorescence	1.75 ± 0.15	2	3.67 ± 0.05	96.0	1.36
			20	23.53 ± 0.72	108.9	3.06
	RGB	1.85 ± 0.13	2	3.79 ± 0.04	97.0	1.06
			20	23.23 ± 1.12	106.9	4.82
Urine sample	Fluorescence	0.10 ± 0.06	2	2.26 ± 0.06	108.0	2.65
			20	20.22 ± 0.62	100.6	3.07
	RGB	0.14 ± 0.05	2	2.15 ± 0.09	100.5	4.19
			20	20.45 ± 0.49	101.6	2.40
Serum	Fluorescence	0.10 ± 0.03	2	2.32 ± 0.05	111.0	2.16
			20	20.12 ± 0.71	100.1	3.53
	RGB	0.10 ± 0.05	2	2.27 ± 0.04	108.5	1.76
			20	20.26 ± 0.65	100.8	3.21

## Data Availability

The original contributions presented in this study are included in the article/Supplementary Material. Further inquiries can be directed to the corresponding authors.

## Supplementary Materials

The following supporting information can be downloaded at: https://www.mdpi.com/article/10.3390/bios16060351/s1, Scheme S1: Synthetic route of probe **XDP**; Figure S1: ^1^H NMR spectra of Compound **3** in CDCl_3_; Figure S2: ^13^C NMR spectra of Compound **3** in CDCl_3_; Figure S3: ^1^H NMR spectra of **XDP** in CDCl_3_; Figure S4: ^13^C NMR spectra of **XDP** in CDCl_3_; Figure S5: Optimization of sensing conditions; Figure S6: Photostability of **XDP** (10 μM) under Xe lamp irradiation in Tris–HCl buffer (10 mM, pH 7.4) containing 50% DMSO. λex = 470 nm; Table S1: Comparison of fluorescent probes for sequential detection of Cu^2+^ and biothiols with **XDP**; Figure S7: Statistical analysis of RGB responses toward Cu^2+^ (a) and GSH (b). (c) Job’s plot for **XDP–Cu^2+^** complex formation, recorded at 605 nm with a constant total concentration of [**XDP**] + [Cu^2+^] = 10 μM; Figure S8: Mass spectrometry spectrum of probe (a) **XDP**, (b) **XDP** + Cu^2+^; Figure S9: Cytotoxicity of XDP toward HeLa cells at different concentrations; Figure S10: Average fluorescence intensity and statistical analysis in HeLa cells and zebrafish; Computational Details. References [39,40,41,42,43,44,45,46,47,48,49,50,51] are cited in the Supplementary Materials.

## References

[1] Hu P., Wang Y., Zhang Y., Jin Y. (2022). Glass Nanopore Detection of Copper Ions in Single Cells Based on Click Chemistry. Anal. Chem..

[2] Zhang S., Mei Y., Liu J., Liu Z., Tian Y. (2024). Alkyne-tagged SERS nanoprobe for understanding Cu^+^ and Cu^2+^ conversion in cuproptosis processes. Nat. Commun..

[3] Zhu J., Graziotto M.E., Cottam V., Hawtrey T., Adair L.D., Trist B.G., Pham N.T.H., Rouaen J.R.C., Ohno C., Heisler M. (2024). Near-Infrared Ratiometric Fluorescent Probe for Detecting Endogenous Cu^2+^ in the Brain. ACS Sens..

[4] Chen L., Min J., Wang F. (2022). Copper homeostasis and cuproptosis in health and disease. Signal Transduct. Target. Ther..

[5] Pezacki A.T., Matier C.D., Gu X., Kummelstedt E., Bond S.E., Torrente L., Jordan-Sciutto K.L., DeNicola G.M., Su T.A., Brady D.C. (2022). Oxidation state-specific fluorescent copper sensors reveal oncogene-driven redox changes that regulate labile copper(II) pools. Proc. Natl. Acad. Sci. USA.

[6] Timoshenko R.V., Gorelkin P.V., Vaneev A.N., Krasnovskaya O.O., Akasov R.A., Garanina A.S., Khochenkov D.A., Iakimova T.M., Klyachko N.L., Abakumova T.O. (2023). Electrochemical Nanopipette Sensor for In Vitro/In Vivo Detection of Cu^2+^ Ions. Anal. Chem..

[7] Falcone E., Vigna V., Schueffl H., Stellato F., Vileno B., Bouraguba M., Mazzone G., Proux O., Morante S., Heffeter P. (2024). When Metal Complexes Evolve, and a Minor Species is the Most Active: The Case of Bis(Phenanthroline)Copper in the Catalysis of Glutathione Oxidation and Hydroxyl Radical Generation. Angew. Chem..

[8] Maiti B.K., Singh M. (2025). Cysteine-based biomolecules regulate cellular copper- and redox-homeostasis. Coord. Chem. Rev..

[9] Falcone E., Ritacca A.G., Hager S., Schueffl H., Vileno B., El Khoury Y., Hellwig P., Kowol C.R., Heffeter P., Sicilia E. (2022). Copper-Catalyzed Glutathione Oxidation is Accelerated by the Anticancer Thiosemicarbazone Dp44mT and Further Boosted at Lower pH. J. Am. Chem. Soc..

[10] Kotuniak R., Strampraad M.J.F., Bossak-Ahmad K., Wawrzyniak U.E., Ufnalska I., Hagedoorn P.L., Bal W. (2020). Key Intermediate Species Reveal the Copper(II)-Exchange Pathway in Biorelevant ATCUN/NTS Complexes. Angew. Chem..

[11] Ufnalska I., Drew S.C., Zhukov I., Szutkowski K., Wawrzyniak U.E., Wróblewski W., Frączyk T., Bal W. (2021). Intermediate Cu(II)-Thiolate Species in the Reduction of Cu(II)GHK by Glutathione: A Handy Chelate for Biological Cu(II) Reduction. Inorg. Chem..

[12] Kartoglu B., Bodur S., Zeydanlı D., Gover T., Ozaydın E., Bakırdere E.G., Bakırdere S. (2023). Determination of copper in rose tea samples using flame atomic absorption spectrometry after emulsification liquid–liquid microextraction. Food Chem..

[13] Trevelin A.M., Vinhal J.O., Viana L.N., Saint’Pierre T.D., Cassella R.J. (2024). Disruption of a three-component solution as a novel strategy for Cu and Ni extraction from vegetable oils for their determination by GF AAS. Food Chem..

[14] Huang Y., Tian J., Wu L., Xuan D., Fang D., Hu G., Qu W., Zhou Y. (2025). Development of a direct dilution method for the simultaneous determination of 40 metal and non-metallic elements in urine and blood samples within 6 min using ICP-MS: Application of comprehensive biomonitoring of elementomic phenotypes of individuals in paired biological samples. Talanta.

[15] Van Acker T., Theiner S., Bolea-Fernandez E., Vanhaecke F., Koellensperger G. (2023). Inductively coupled plasma mass spectrometry. Nat. Rev. Methods Primers.

[16] Tian Y., Liu J., Qiao J., Ge F., Yang Y., Zhang Q. (2025). Advancements in Electrochemical Sensing Technology for Heavy Metal Ions Detection. Food Chem. X.

[17] Sun S., Song P., Sun C., Wang J. (2023). A novel electrochemical sensor based on MOFs composites, gold nanoparticles, and DNAzyme for Cu^2+^ sensitive detection. J. Environ. Chem. Eng..

[18] Liu X., Shi T., Xu C., Zhu M., Wang Y. (2023). A highly selective and sensitive ICT-based Cu^2+^ fluorescent probe and its application in bioimaging. Ecotoxicol. Environ. Saf..

[19] Zhou H., Wang S., Jin Y., Pang X.-F., Zhao Q., Zhang T., Zhang J. (2025). A near-infrared “turn-on” fluorescent probe for selective detection of copper(II) ions in aqueous media and its application in cell imaging. Spectrochim. Acta Part A.

[20] Ma X., Lin S., Dang Y., Dai Y., Zhang X., Xia F. (2019). Carbon dots as an “on-off-on” fluorescent probe for detection of Cu(II) ion, ascorbic acid, and acid phosphatase. Anal. Bioanal. Chem..

[21] Ning G., Li B., Liu J., Xiao Q., Huang S. (2022). Red-emission carbon dots as fluorescent “on–off–on” probe for highly sensitive and selective detection of Cu^2+^ and glutathione. Anal. Bioanal. Chem..

[22] Yang J., Chen W., Chen X., Zhang X., Zhou H., Du H., Wang M., Ma Y., Jin X. (2021). Detection of Cu^2+^ and S^2−^ with fluorescent polymer nanoparticles and bioimaging in HeLa cells. Anal. Bioanal. Chem..

[23] Wang Z.-G., Ding X.-J., Huang Y.-Y., Yan X.-J., Ding B., Li Q.-Z., Xie C.-Z., Xu J.-Y. (2020). The development of coumarin Schiff base system applied as highly selective fluorescent/colorimetric probes for Cu^2+^ and tumor biomarker glutathione detection. Dye. Pigment..

[24] Ding C., Ren T.B. (2023). Near infrared fluorescent probes for detecting and imaging active small molecules. Coord. Chem. Rev..

[25] Sun Q., He D., Zhang L., Li Z., Qu L., Sun Y. (2023). Coumarin-hemicyanine-based far-red to near-infrared fluorescent probes: A new generation of fluorescent probe design platform. Trends Anal. Chem..

[26] Kumar G.D., Banasiewicz M., Poronik Y.M., Morawski O., Deperasinska I., Gryko D.T. (2026). Synthesis and Photophysical Properties of Polarized 7-Heteroaryl Coumarins. J. Org. Chem..

[27] Campos L.A.D., García-Río L., Affeldt R.F., Gerola A.P. (2024). A fluorescent probe based on scopoletin-3-carboxylic acid: pH and micellization evaluation. Spectrochim. Acta Part A Mol. Biomol. Spectrosc..

[28] Ren T.B., Wang Z.Y., Xiang Z., Lu P., Lai H.H., Yuan L., Zhang X.B., Tan W. (2020). A General Strategy for Development of Activatable NIR-II Fluorescent Probes for In Vivo High-Contrast Bioimaging. Angew. Chem..

[29] Fu S., Deng L., Xue L., Gao Y., Cheng Y., Wang H. (2025). A coumarin based “turn-on” type fluorescent probe for the detection of Cu^2+^ with mechanochromic property. J. Mol. Struct..

[30] Gao Y., Deng L., Xue L., Cheng Y., Fu S., Wang H. (2025). A Novel Coumarin Based Fluorescent Probe for Cu^2+^ Detection as well as Applications. J. Fluoresc..

[31] Zhou H., Sun H., Zhang Z., Fang X., Bu S., Li C., Wan Y., Li N., Hao Z., Ma C. (2026). A highly sensitive colorimetric platform based on tricoordinate metal MOFs nanoenzymes and smartphone RGB–assisted analysis for visualized detection of Salmonella typhimurium. Anal. Bioanal. Chem..

[32] Wu J., Li R., Liu S. (2022). A novel dual-emission fluorescent probe for ratiometric and visual detection of Cu^2+^ ions and Ag^+^ ions. Anal. Bioanal. Chem..

[33] Lv J., Jiao X., He D.D., Hussain E., Yang N., Wang Y., Zhang H., Chen L., Jin X., Liu N. (2023). Sensitive and discriminative detection of cysteine by a Nile red-based NIR fluorescence probe. Anal. Bioanal. Chem..

[34] Ouyang J., Sun L., Zeng Z., Zeng C., Zeng F., Wu S. (2019). Nanoaggregate Probe for Breast Cancer Metastasis through Multispectral Optoacoustic Tomography and Aggregation-Induced NIR-I/II Fluorescence Imaging. Angew. Chem..

[35] Zhu L., Song G., Zhang W., Wu Y., Chen Y., Song J., Wang D., Li G., Tang B.Z., Li Y. (2025). Aggregation induced emission luminogen bacteria hybrid bionic robot for multimodal phototheranostics and immunotherapy. Nat. Commun..

[36] Lv H., Li H., Xin L., Guo F. (2020). Effect of stepwise protonation of an N-containing ligand on the formation of metal–organic salts and coordination complexes in the solid state. CrystEngComm.

[37] Gu Y., Sun Y., Zheng W. (2023). Novel strategy for copper precipitation from cupric complexes wastewater: Catalytic oxidation or reduction self-decomplexation?. J. Hazard. Mater..

[38] Lystrom L., Roberts A., Dandu N., Kilina S. (2021). Surface-Induced Deprotonation of Thiol Ligands Impacts the Optical Response of CdS Quantum Dots. Chem. Mater..

[39] Zhao Y., Ma Q., Jiang Y., Liang X., Wang S., Zhao S., Li W., Yang X. (2025). A coumarin-based fluorescent probe for sequential detection of Cu^2+^ and GSH with its application in visual sensing. Microchem. J..

[40] Kuang C., Li Y., Zhang X., Wang J., Zhao S., Sun Y., Li M. (2023). A mitochondria-targeted phosphorescent probe for sequentially detecting Cu^2+^ and cysteine and its imaging in living cells and in vivo. Dye. Pigment..

[41] Bayindir S., Akar S. (2024). Synthesis of Phenol-Hydrazide-Appended Tetraphenylethenes as Novel On–Off–On Cascade Sensors of Copper and Glutathione. ACS Omega.

[42] Shabbir A., Shahzad S.A., Alzahrani A.Y.A., Khan Z.A., Yar M., Rauf W. (2025). A Multimode fluorescent sensor for sequential detection of Cu^2+^ and cysteine as well as pH sensor with real sample Applications: Extensive experimental and DFT studies. Spectrochim. Acta Part A.

[43] Jung S., Gil D., Kim C. (2023). A New Chromone-based Sequential Functioning Colorimetric Chemosensor for Cu^2+^ and Cysteine in Near-perfect Aqueous Media. Asian J. Org. Chem..

[44] Wang L., Chen Y., Xing Z., Wang L., Ma J. (2025). A novel carbazolyl thiophene-based fluorescent “off-on” probe PCTMF for sequential detection of Cu^2+^ and cysteine in aqueous. J. Mol. Struct..

[45] Wang M., Li S., Shi J., Liu Y., Cao D., Zhao L. (2023). A simple BODIPY-based fluorescent probe for sequential recognition of Cu^2+^ and GSH and its application on test strips and bioimaging in living cells. J. Mol. Struct..

[46] Suganthirani K., Thiruppathiraja T., Lakshmipathi S., Malecki J.G., Murugesapandian B. (2025). Aminothiophenol and 7-diethylamino-4-hydroxycoumarin derived probe for reversible turn off–on–off detection of Cu^2+^ ions and cysteine. Spectrochim. Acta Part A Mol. Biomol. Spectrosc..

[47] Pang S., Yu Y., Wu W., You J., Liang Y., Wu C., Li B. (2025). Synthesis and Applications of a Novel Naphthalimide-Based Fluorescent Probe for Relay Recognition of Cu^2+^ and Cysteine. Luminescence.

[48] Shi Y., Yu J., Song Y., Fan J., Wang X., Li S., Li H. (2025). Multifunctional near-infrared fluorescent probe for sensing of lysine and Cu^2+^/Fe^3+^ and relay detection of biothiols. Talanta.

[49] Liu L., Liu B., Hao Y., Wang J., Xu X., Shang X. (2024). Theory and experiment: The synthesis and drug application of “ON-OFF-ON” fluorescent probes for copper and biothiols detection. J. Pharm. Biomed. Anal..

[50] Dong Y., Qiao R., Song Q., Chen M., Wang S., Hu J., Wang X., Xue W., Zhang Y., Bai C. (2025). Two birds with one stone: A near-infrared AIE fluorescent probe for highly selective recognition of Cu^2+^/GSH and bioimaging. J. Photochem. Photobiol. A Chem..

[51] Frisch M.J., Trucks G.W., Schlegel H.B., Scuseria G.E., Robb M.A., Cheeseman J.R., Scalmani G., Barone V., Mennucci B., Petersson G.A. (2009). Gaussian 09.

