# Supplementary material for: A Coumarin-Based Probe for Sequential ON–OFF–ON Detection of Cu2+ and Biothiols: Naked-Eye Detection, Smartphone RGB Readout and In Vivo Imaging
Source: Biosensors (Basel). 2026 Jun 22;16(6):351. doi: 10.3390/bios16060351 (PMC13297486; doi:10.3390/bios16060351)
Supplement: Supplementary file 1 [file biosensors-16-00351-s001.zip › biosensors-4360033-supplementary.pdf]

Supplementary Information

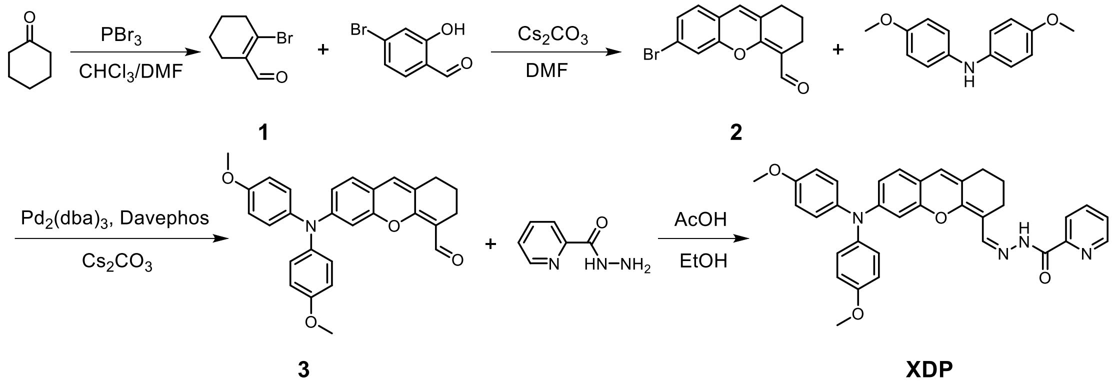

Scheme S1. Synthetic route of probe **XDP**.

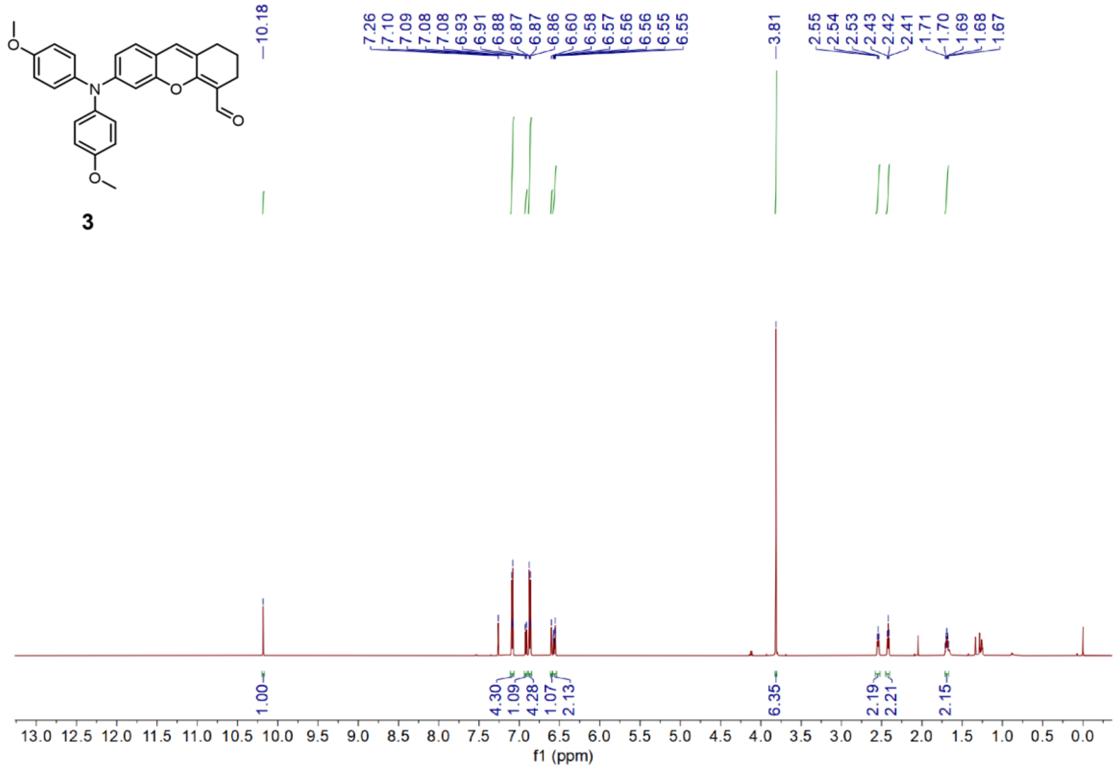

Figure S1. ^{1}H NMR spectra of Compound **3** in $CDCl_3$.

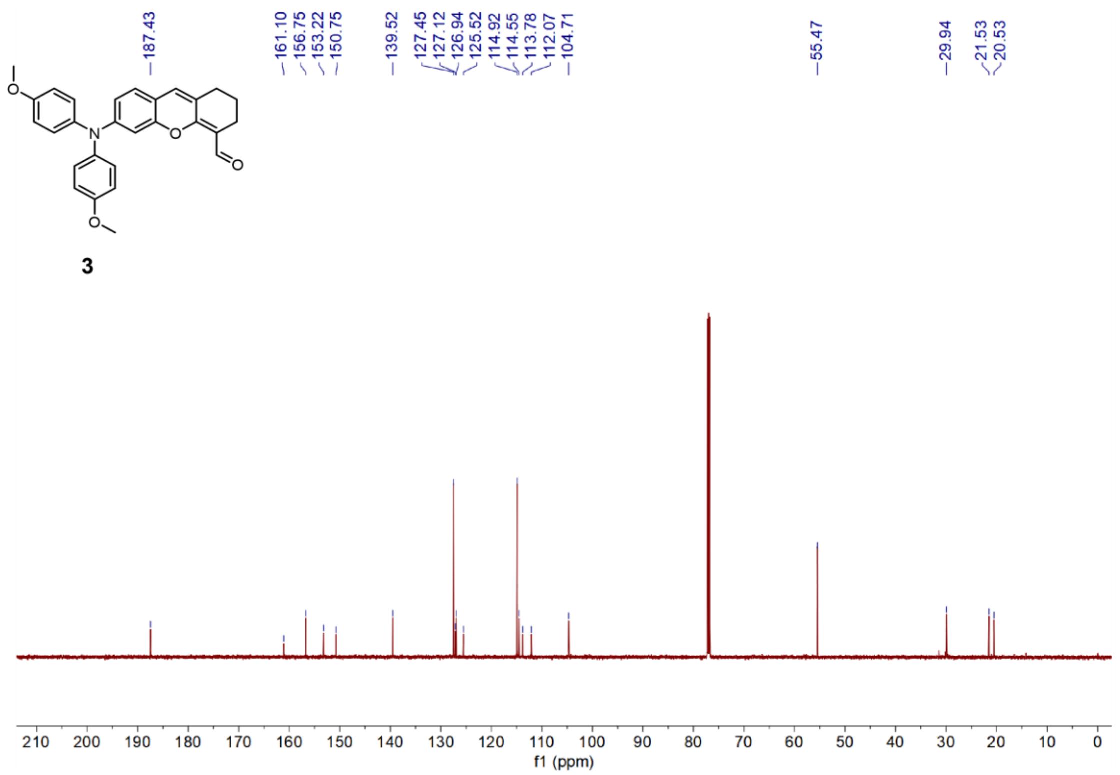

Figure S2. ^{13}C NMR spectra of Compound **3** in $CDCl_3$.

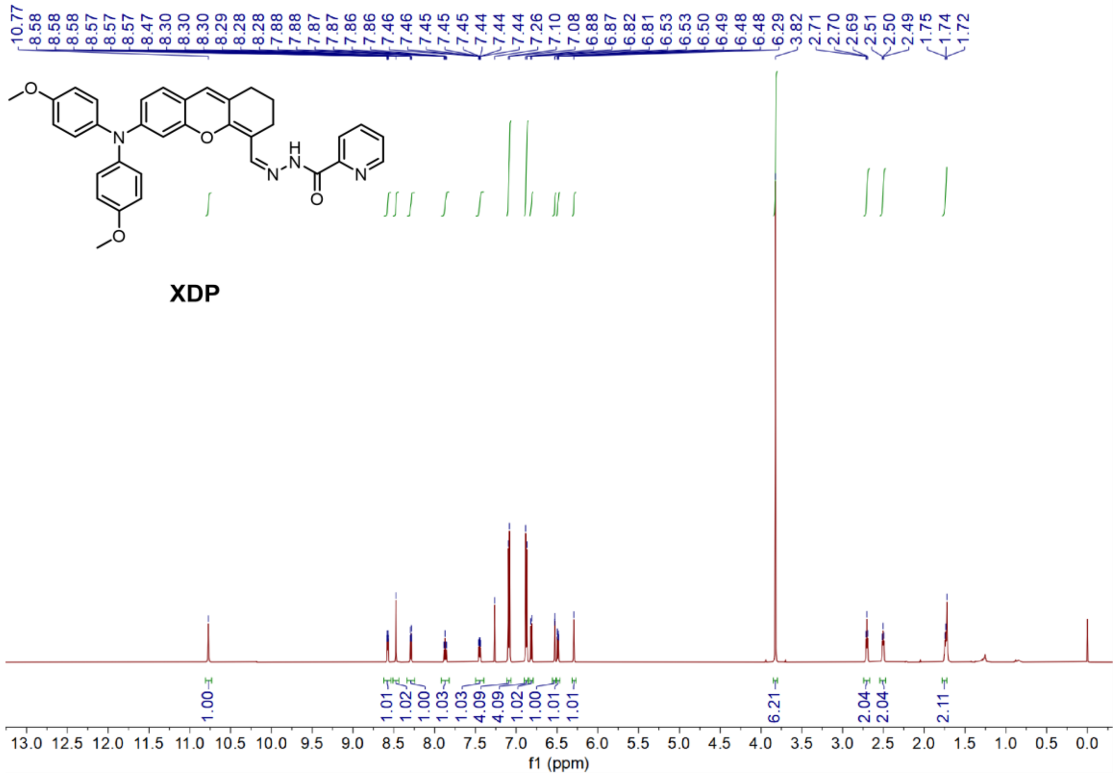

Figure S3. ^{1}H NMR spectra of **XDP** in $CDCl_3$.

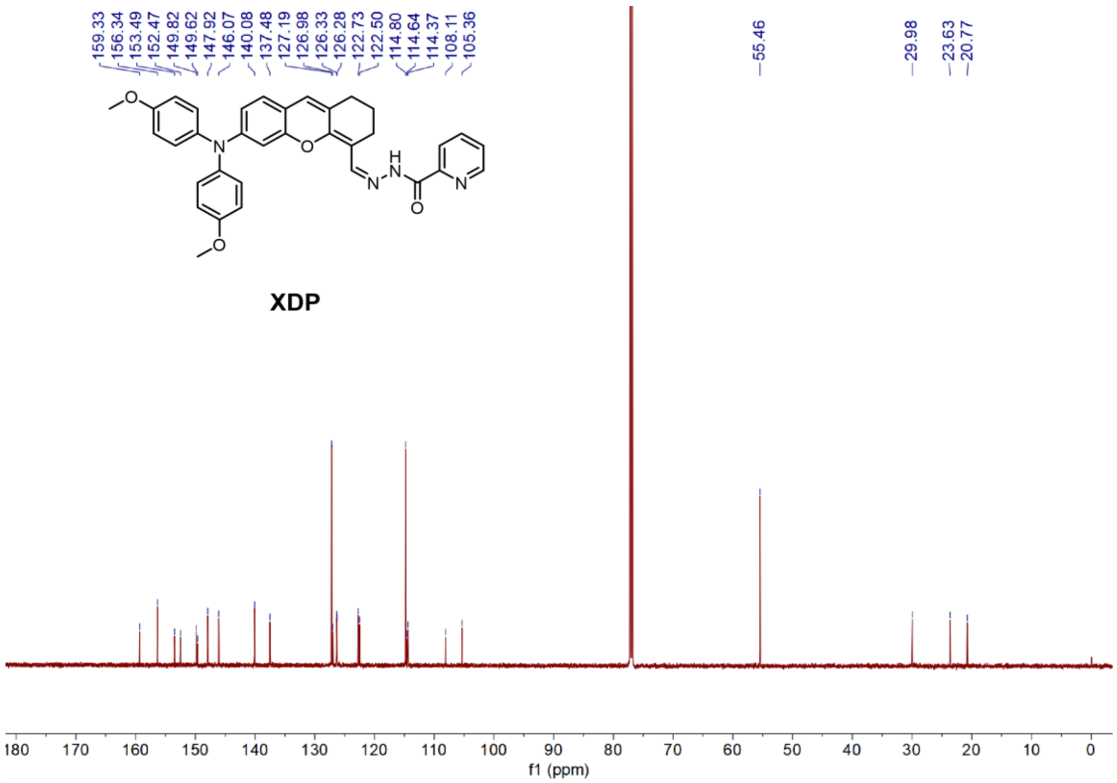

Figure S4. ^{13}C NMR spectra of **XDP** in $CDCl_3$.

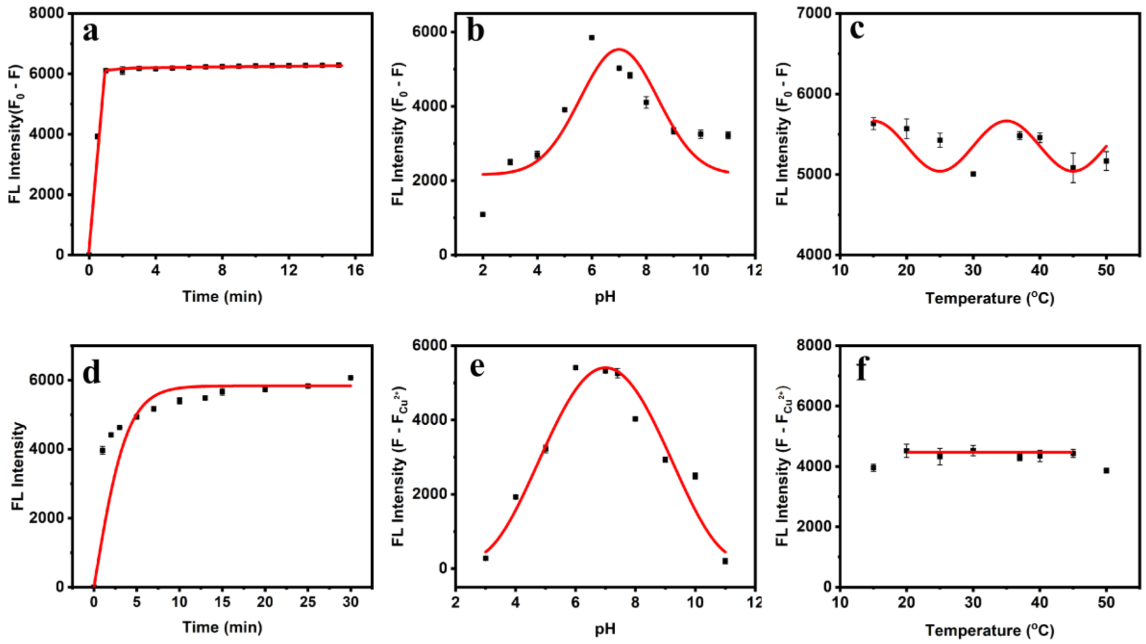

Figure S5. Optimization of sensing conditions. Results are averaged over three independent runs. (a–c) Cu^{2+} (10 μM) detection: time dependence (**a**), pH effect (**b**), and temperature effect (**c**). (**d–f**) GSH (30 μM) detection in the **XDP–Cu^{2+}** system (10 μM): time dependence (**d**), pH effect (**e**), and temperature effect (**f**).

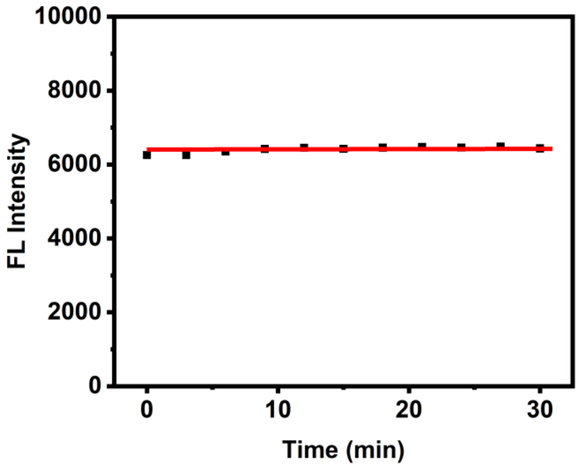

Figure S6. Photostability of **XDP** (10 μM) under Xe lamp irradiation in Tris–HCl buffer (10 mM, pH 7.4) containing 50% DMSO. λex = 470 nm.

Table S1. Comparison of fluorescent probes for sequential detection of Cu^{2+} and biothiols with **XDP**.

Probe	Linear range (μM)	LOD (μM)	Time (min)	Detection mode	Application
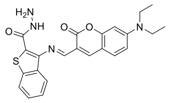	Cu^{2+}: 0.1–0.7<br>GSH: 0–15	Cu^{2+}: 0.207<br>GSH: 0.43	Cu^{2+}: 0.33<br>GSH: 0.66	Fluorescence, Smartphone-Assisted RGB analysis	Test papers, Food and textile sample[39]
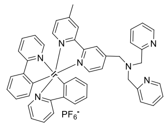	Cu^{2+}: 0–16<br>Cys: 20–200	Cu^{2+}: 0.04<br>Cys: 1.21	None	Fluorescence	Cells, Zebrafish, Water and serum samples[40]
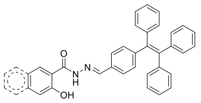	Cu^{2+}: 0–3<br>GSH: 0–4	Cu^{2+}:0.747 (TPE Ⅰ), 0.597 (TPE Ⅱ)<br>GSH: 0.54	Cu^{2+}: 2.0	Fluorescence	Water, Filter paper[41]
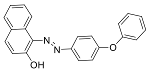	Cu^{2+}: 0–1<br>Cys: 0–100	Cu^{2+}: 13<br>Cys: 0.084	Cu^{2+}:1.5	Fluorescence	Tap water, Milk, Soil industrial waste fertilizers[42]
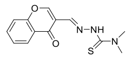	Cu^{2+}: 0–4<br>Cys: 0–80	Cu^{2+}: 0.30<br>Cys: 6.62	None	Uv-vis	Water samples, Test strips[43]
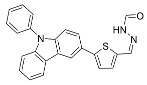	Cu^{2+}: 0–3<br>Cys: 1.5–5.5	Cu^{2+}: 0.14<br>Cys: 0.052	Cu^{2+}: 20	Fluorescence	Test paper[44]
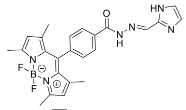	Cu^{2+}: 0–5<br>GSH: 0–59	Cu^{2+}: 0.65<br>GSH: 0.02	None	Fluorescence	Cells, Test strips[45]
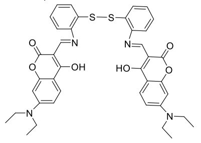	Cu^{2+}: 0–50<br>Cys: 0–60	Cu^{2+}: 9.7×10^{-4}<br>Cys: 1.64	None	Fluorescence	Water samples[46]
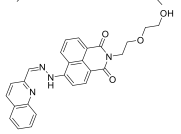	Cu^{2+}: 0–10<br>Cys: 0–20	Cu^{2+}: 3.6×10^{-2}<br>Cys: 4.2×10^{-2}	Cu^{2+}: 0.2<br>Cys: 9.0	Fluorescence	Test strip, Milk[47]
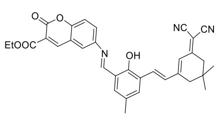	Cu^{2+}: 2–20<br>Biothiols: 2–20	Cu^{2+}: 1.70<br>GSH: 0.30<br>Cys: 1.10<br>Hcy: 1.90	Cu^{2+}: 2.5	Fluorescence	Cells[48]

Probe	Linear range (μM)	LOD (μM)	Time (min)	Detection mode	Application
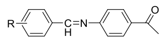	None	Cys: 34<br>GSH: 20	Cu^{2+}: 5.0<br>GSH: 10<br>Hcy and Cys: 6.0	Fluorescence	Cells [49]
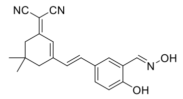	Cu^{2+}: 1–20<br>GSH: 1–100	Cu^{2+}: 0.0353<br>GSH: 0.347	GSH: 4.0	Fluorescence Smartphone-Assisted RGB analysis	Cell, Water samples, Mice[50]
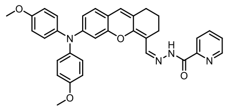	Cu^{2+}: 1–80<br>GSH: 1 – 15<br>Cys: 1 – 30<br>Hcy: 1 – 20	Cu^{2+}: 0.108<br>GSH: 0.116<br>Cys: 0.342<br>Hcy: 0.233	Cu^{2+}: 2.0<br>Biothiols: 15.0	Fluorescence, Naked-eye visualization, Smartphone-Assisted RGB analysis	Water samples, Cells, Zebrafish (This work)

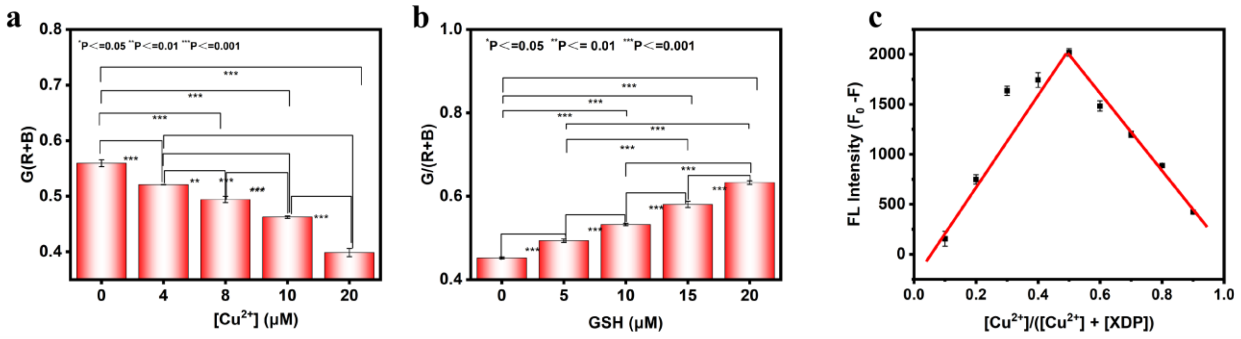

Figure S7. Statistical analysis of RGB responses toward Cu^{2+} (a) and GSH (b). (c) Job's plot for **XDP–Cu**$^{2+}$ complex formation, recorded at 605 nm with a constant total concentration of [**XDP**] + [Cu^{2+}] = 10 μM.

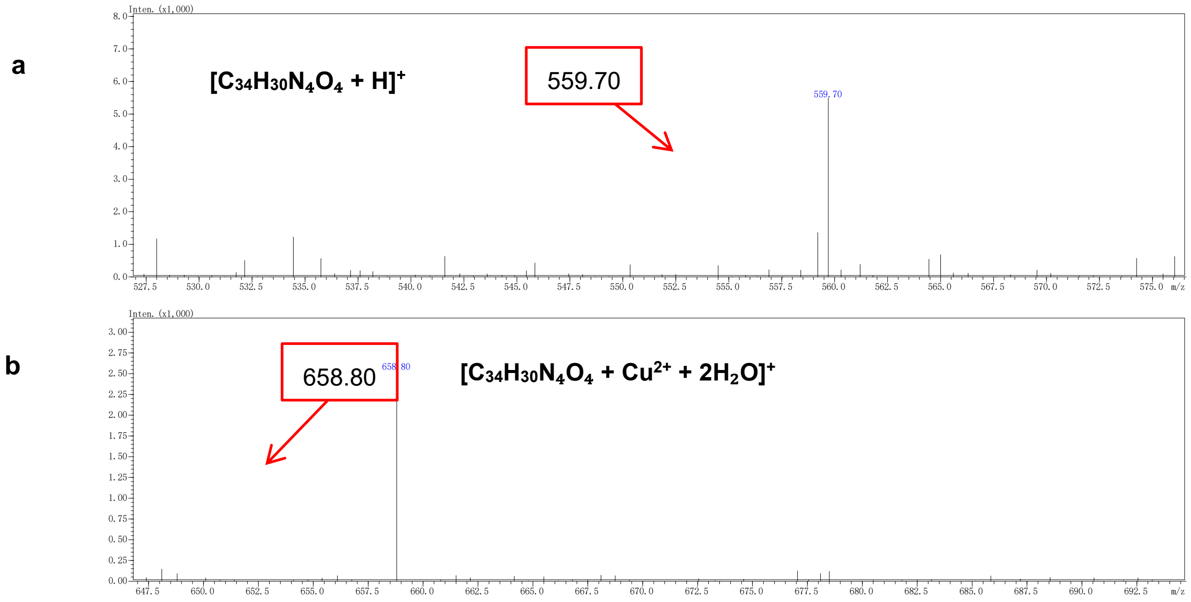

Figure S8. Mass spectrometry spectrum of probe (**a**) **XDP**, (**b**) **XDP** + Cu^{2+}.

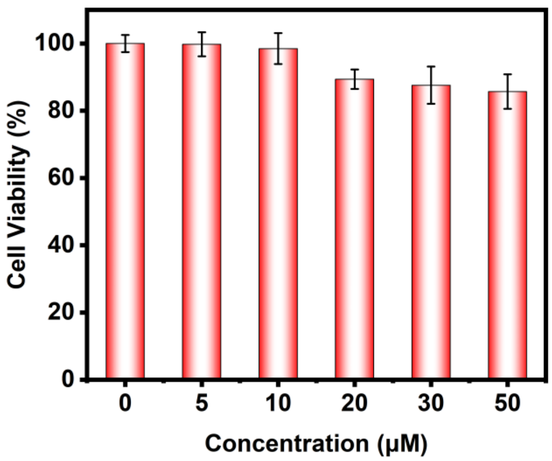

Figure S9. Cytotoxicity of **XDP** toward HeLa cells at different concentrations.

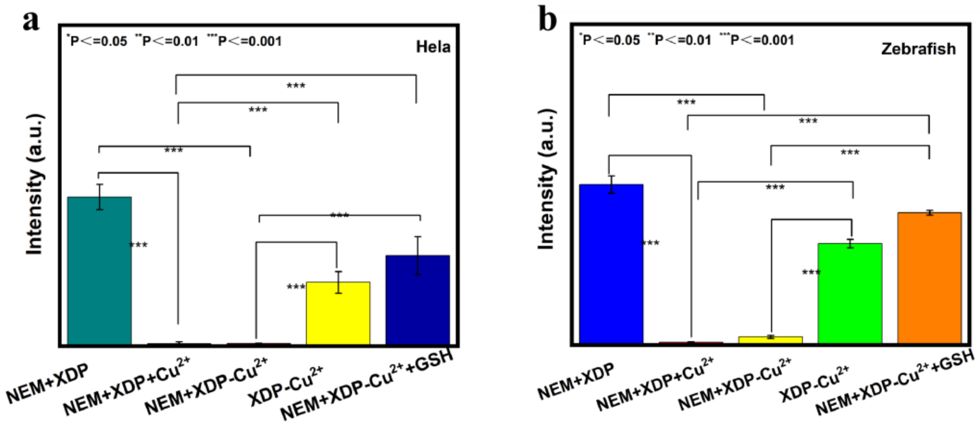

Figure S10. Average fluorescence intensity and statistical analysis in HeLa cells and zebrafish.

Computational Details

All quantum chemical calculations were performed using Gaussian 09 software [51]. Ground-state geometry optimization was carried out using density functional theory (DFT) at the B3LYP/6-31+G(d) level. For the Cu^{2+} coordination system, unrestricted calculations were employed to describe its open-shell electronic characteristics. Solvent effects in the aqueous environment were simulated using the SMD continuum solvation model.

References

39. Zhao, Y.; Ma, Q.; Jiang, Y.; Liang, X.; Wang, S.; Zhao, S.; Li, W.; Yang, X. A coumarin-based fluorescent probe for sequential detection of Cu^{2+} and GSH with its application in visual sensing. *Microchem. J.* **2025**, *215*, 114552. https://doi.org/10.1016/j.microc.2025.114552.

40. Kuang, C.; Li, Y.; Zhang, X.; Wang, J.; Zhao, S.; Sun, Y.; Li, M. A mitochondria-targeted phosphorescent probe for sequentially detecting Cu^{2+} and cysteine and its imaging in living cells and in vivo. *Dyes Pigm.* **2023**, *220*, 111702. https://doi.org/10.1016/j.dyepig.2023.111702.

41. Bayindir, S.; Akar, S. Synthesis of Phenol-Hydrazide-Appended Tetraphenylethenes as Novel On–Off–On Cascade Sensors of Copper and Glutathione. *ACS Omega* **2024**, *9*, 26257–26266. https://doi.org/10.1021/acsomega.4c02043.

42. Shabbir, A.; Shahzad, S.A.; Alzahrani, A.Y.A.; Khan, Z.A.; Yar, M.; Rauf, W. A Multimode fluorescent sensor for sequential detection of Cu^{2+} and cysteine as well as pH sensor with real sample Applications: Extensive experimental and DFT studies.

Spectrochim. Acta Part A **2025**, *327*, 125414. https://doi.org/10.1016/j.saa.2024.125414.

43. Jung, S.; Gil, D.; Kim, C. A New Chromone-based Sequential Functioning Colorimetric Chemosensor for Cu^{2+} and Cysteine in Near-perfect Aqueous Media. *Asian J. Org. Chem.* 2023, 12, e202300151. https://doi.org/10.1002/ajoc.202300151
44. Wang, L.; Chen, Y.; Xing, Z.; Wang, L.; Ma, J. A novel carbazolyl thiophene-based fluorescent "off-on" probe PCTMF for sequential detection of Cu^{2+} and cysteine in aqueous. *J. Mol. Struct.* **2025**, *1321*, 140268. https://doi.org/10.1016/j.molstruc.2024.140268.
45. Wang, M.; Li, S.; Shi, J.; Liu, Y.; Cao, D.; Zhao, L. A simple BODIPY-based fluorescent probe for sequential recognition of Cu^{2+} and GSH and its application on test strips and bioimaging in living cells. *J. Mol. Struct.* **2023**, *1294*, 136393. https://doi.org/10.1016/j.molstruc.2023.136393.
46. Suganthirani, K.; Thiruppathiraja, T.; Lakshmipathi, S.; Malecki, J.G.; Murugesapandian, B. Aminothiophenol and 7-diethylamino-4-hydroxycoumarin derived probe for reversible turn off–on–off detection of Cu^{2+} ions and cysteine. *Spectrochim. Acta Part A: Mol. Biomol. Spectrosc.* **2025**, *327*, 125315. https://doi.org/10.1016/j.saa.2024.125315.
47. Pang, S.; Yu, Y.; Wu, W.; You, J.; Liang, Y.; Wu, C.; Li, B. Synthesis and Applications of a Novel Naphthalimide-Based Fluorescent Probe for Relay Recognition of Cu^{2+} and Cysteine. *Luminescence* **2025**, *40*, e70173. https://doi.org/10.1002/bio.70173.
48. Shi, Y.; Yu, J.; Song, Y.; Fan, J.; Wang, X.; Li, S.; Li, H. Multifunctional near-infrared fluorescent probe for sensing of lysine and Cu^{2+}/Fe^{3+} and relay detection of biothiols. *Talanta* **2025**, *281*, 126944. https://doi.org/10.1016/j.talanta.2024.126944.
49. Liu, L.; Liu, B.; Hao, Y.; Wang, J.; Xu, X.; Shang, X. Theory and experiment: The synthesis and drug application of "ON-OFF-ON" fluorescent probes for copper and biothiols detection. *J. Pharm. Biomed. Anal.* **2024**, *239*, 115876. https://doi.org/10.1016/j.jpba.2023.115876.
50. Dong, Y.; Qiao, R.; Song, Q.; Chen, M.; Wang, S.; Hu, J.; Wang, X.; Xue, W.; Zhang, Y.; Bai, C.; et al. Two birds with one stone: A near-infrared AIE fluorescent probe for highly selective recognition of Cu^{2+}/GSH and bioimaging. *J. Photochem. Photobiol. A: Chem.* **2025**, *462*, 116263. https://doi.org/10.1016/j.jphotochem.2025.116263.
51. Frisch, M. J.; Trucks, G. W.; Schlegel, H. B.; Scuseria, G. E.; Robb, M. A.; Cheeseman, J. R.; Scalmani, G.; Barone, V.; Mennucci, B.; Petersson, G. A.; et al. Gaussian 09, Revision D.01, Gaussian, Inc., Wallingford CT, 2009.
